# PGC-1α and PGC-1β Increase Protein Synthesis via ERRα in C2C12 Myotubes

**DOI:** 10.3389/fphys.2018.01336

**Published:** 2018-09-25

**Authors:** Erin L. Brown, Victoria C. Foletta, Craig R. Wright, Patricio V. Sepulveda, Nicky Konstantopoulos, Andrew Sanigorski, Paul Della Gatta, David Cameron-Smith, Anastasia Kralli, Aaron P. Russell

**Affiliations:** ^1^Institute for Physical Activity and Nutrition, School of Exercise and Nutrition Sciences, Deakin University, Burwood, VIC, Australia; ^2^School of Medicine, Deakin University, Waurn Ponds, VIC, Australia; ^3^Liggins Institute, University of Auckland, Auckland, New Zealand; ^4^Department of Physiology, School of Medicine, Johns Hopkins University, Baltimore, MD, United States

**Keywords:** PGC-1α, PGC-1β, ERRα, protein synthesis, C2C12 myotubes, muscle mass, metabolism, resistance exercise

## Abstract

The transcriptional coactivators peroxisome proliferator-activated receptor-γ coactivator-1α (PGC-1α) and PGC-1β are positive regulators of skeletal muscle mass and energy metabolism; however, whether they influence muscle growth and metabolic adaptations via increased protein synthesis is not clear. This study revealed PGC-1α or PGC-1β overexpression in C2C12 myotubes increased protein synthesis and myotube diameter under basal conditions and attenuated the loss in protein synthesis following the treatment with the catabolic agent, dexamethasone. To investigate whether PGC-1α or PGC-1β signal through the Akt/mTOR pathway to increase protein synthesis, treatment with the PI3K and mTOR inhibitors, LY294002 and rapamycin, respectively, was undertaken but found unable to block PGC-1α or PGC-1β’s promotion of protein synthesis. Furthermore, PGC-1α and PGC-1β decreased phosphorylation of Akt and the Akt/mTOR substrate, p70S6K. In contrast to Akt/mTOR inhibition, the suppression of ERRα, a major effector of PGC-1α and PGC-1β activity, attenuated the increase in protein synthesis and myotube diameter in the presence of PGC-1α or PGC-1β overexpression. To characterize further the biological processes occurring, gene set enrichment analysis of genes commonly regulated by both PGC-1α and PGC-1β was performed following a microarray screen. Genes were found enriched in metabolic and mitochondrial oxidative processes, in addition to protein translation and muscle development categories. This suggests concurrent responses involving both increased metabolism and myotube protein synthesis. Finally, based on their known function or unbiased identification through statistical selection, two sets of genes were investigated in a human exercise model of stimulated protein synthesis to characterize further the genes influenced by PGC-1α and PGC-1β during physiological adaptive changes in skeletal muscle.

## Introduction

The significant reduction in skeletal muscle mass known as atrophy occurs as a consequence of protein catabolism outweighing protein synthesis rates (reviewed in Favier et al., 2008; Huang and Zhu, 2016) with mitochondrial dysfunction potentially contributing to muscle atrophy (Romanello et al., 2010; Russell et al., 2014). Severe muscle atrophy can lead to debilitating consequences including loss in strength, mobility, and independence, and increased morbidity and mortality as an outcome from sustained inactivity, increasing age, and systemic diseases (reviewed in Kalyani et al., 2014). The transcriptional coactivators, peroxisome proliferator-activated receptor-γ coactivator-1α (PGC-1α) and PGC-1β, contribute to the control of skeletal muscle mass and energy metabolism through their coordinated transcriptional regulation of genes involved in mitochondrial biogenesis and fusion (Wu et al., 1999; St-Pierre et al., 2003; Schreiber et al., 2004; Soriano et al., 2006; Liesa et al., 2008; Shao et al., 2010), protection against protein degradation (Brault et al., 2010), skeletal muscle fiber type determination (Lin et al., 2002; Arany et al., 2007; Rasbach et al., 2010), glucose transport (Michael et al., 2001; Kelly et al., 2009), creatine uptake (Brown et al., 2014), lipid oxidation (Benton et al., 2008; Espinoza et al., 2010), and angiogenesis (Arany et al., 2008; Rowe et al., 2011). The expression of PGC-1α and/or PGC-1β is reduced in conditions associated with skeletal muscle atrophy and perturbed metabolic function, such as aging (Ling et al., 2004; Rodriguez-Calvo et al., 2006; Joseph et al., 2012), denervation (Sandri et al., 2006; Sacheck et al., 2007), heart failure (Garnier et al., 2003; Vescovo et al., 2005), sepsis (Menconi et al., 2010), cancer cachexia and renal failure (Sandri et al., 2006), amyotrophic lateral sclerosis (ALS; Russell et al., 2013), insulin-resistance, and diabetes (Mootha et al., 2003; Patti et al., 2003).

To extend these findings, the overexpression of PGC-1α or PGC-1β in mouse skeletal muscle was found protective against muscle mass loss following denervation, fasting, and hindlimb unloading (Sandri et al., 2006; Brault et al., 2010; Cannavino et al., 2014, 2015) and in a model of Duchenne muscular dystrophy (DMD; Handschin et al., 2007; Selsby et al., 2012). Furthermore, PGC-1α attenuated muscle atrophy by maintaining mitochondrial biogenesis during the on-set and progression of ALS (Da Cruz et al., 2012). One mechanism known to cause a decrease in protein degradation following PGC-1α or PGC-1β overexpression is via the attenuation of FOXO3a transcriptional activity (Sandri et al., 2006; Brault et al., 2010). In addition, PGC-1α prevented hindlimb unloading atrophy by reducing autophagy, proteasome degradation, and maintaining mitochondrial dynamics (Cannavino et al., 2014, 2015). Previously, PGC-1α and PGC-1β overexpression *in vitro* has been suggested not to increase protein synthesis (Brault et al., 2010); however, rodent and cell culture studies identified a specific isoform of PGC-1α, PGC-1α4, to stimulate muscle hypertrophy via induction of insulin-like growth factor-1 (IGF-1) and repression of myostatin (Ruas et al., 2012), positive and negative regulators of protein synthesis, respectively (Taylor et al., 2001; Quinn et al., 2007). In humans, it is unclear whether specific or all isoforms of PGC-1α promote a hypertrophic response to either acute resistant or endurance training (Ydfors et al., 2013; Lundberg et al., 2014). Therefore, a clear role for PGC-1α and PGC-1β in regulating skeletal muscle protein synthesis requires further investigation to understand their contribution to muscle fiber hypertrophy and metabolic adaptations.

Many of the PGC-1α and/or PGC-1β-mediated effects on skeletal muscle involve their activation of the orphan nuclear receptor, estrogen-related receptor alpha (ERRα). ERRα is required for PGC-1α and/or PGC-1β to increase the expression of several genes encoding proteins involved in oxidative phosphorylation and mitochondrial biogenesis (Mootha et al., 2004; Schreiber et al., 2004; Shao et al., 2010), mitochondrial fusion (Russell et al., 2013), fatty acid oxidation (Kamei et al., 2003; Schreiber et al., 2004), oxidative muscle fiber phenotype (Rasbach et al., 2010), and angiogenesis (Arany et al., 2008; Rowe et al., 2011) in skeletal muscle. While a role in skeletal muscle differentiation and regeneration has been described for ERRα (LaBarge et al., 2014), its impact on skeletal muscle atrophy and effects on protein turnover under normal or stress conditions have not been well explored (reviewed in Huss et al., 2015). Therefore, whether PGC-1α or PGC-1β can regulate protein synthesis via the ERRα transcriptional pathway remains to be established.

The present study aimed to determine whether PGC-1α and/or PGC-1β can regulate protein synthesis in mouse C2C12 myotubes under basal or catabolic conditions, and if this is mediated via Akt/mTOR signaling, known stimulators of protein synthesis in skeletal muscle (Bodine et al., 2001; Rommel et al., 2001), or ERRα. Additionally, large-scale gene expression profiling followed by bioinformatics analyses were used to identify novel PGC-1α and PGC-1β regulated targets that may influence muscle protein synthesis. Both an unbiased and a biased selection of gene targets from the microarray data were measured in skeletal muscle from a human model of stimulated protein synthesis, acute resistance exercise, in order to further characterize downstream genes influenced by PGC-1α and PGC-1β during physiological changes to muscle mass.

## Materials and Methods

### Cell Culture

Mouse C2C12 myoblasts (American type culture collection; ATCC, Manassas, VA, United States) were incubated at 37°C with 5% CO_2_, in 25 mM glucose Dulbecco’s Modified Eagle Medium (DMEM) supplemented with 10% fetal bovine serum and 1% penicillin streptomycin (PS) (Invitrogen, Carlsbad, CA, United States). Myoblasts were plated at a density of 1.5 × 10^4^/cm^2^ in 12-well plates. Upon confluence (∼48 h), medium was changed to 25 mM DMEM supplemented with 2% horse serum (Invitrogen) and 1% PS for 4–5 days until multinucleated myotubes had formed. Medium was replaced every 48 h.

Myotubes were infected with adenoviruses expressing green fluorescent protein (GFP), PGC-1α or PGC-1β using a multiplicity of infection (MOI) of 100. Cells were harvested after 72 h for RNA, protein, and protein synthesis assays. For experiments using ERRα knockdown, myotubes were infected with an adenovirus expressing shRNA for ERRα (AdshERRα) or its control, AdSUPER, using an MOI 200 (Schreiber et al., 2003). After 24 h, myotubes were infected with an additional dose of AdshERRα (or its control vector), as well as GFP, PGC-1α, or PGC-1β at an MOI of 100. Cells were harvested after 96 h for RNA, protein, and protein synthesis assays. For experiments overexpressing a constitutively active ERRα, myotubes were infected with an adenovirus expressing ERRα with a heterogeneous strong transcriptional activation domain, VP16-ERRα, or its control VP16-control, using an MOI of 100 (Schreiber et al., 2004). Cells were harvested after 72 h for RNA, protein, and protein synthesis assays. Following infection with GFP, PGC-1α, or PGC-1β adenoviruses, myotubes were treated with 10 μM dexamethasone (DEX), 10 μM LY-294002, or 20 ng/mL rapamycin, which were obtained from Sigma-Aldrich (St Louis, MO, United States), and their respective vehicle controls, for 24 h.

### Protein Synthesis

Protein synthesis was determined by measuring the incorporation of [^3^H]-tyrosine (Perkin Elmer, Boston, MA, United States) into the myotubes (modified from Plaisance et al., 2008). Following adenoviral infections, myotubes were incubated in 1 μCi/mL of [^3^H]-tyrosine and 2 mM L-tyrosine (Sigma-Aldrich). After 24 h, cells were washed with cold phosphate buffered saline (PBS), and proteins were harvested and precipitated in 10% trichloroacetic acid (Sigma-Aldrich) on ice for one hour. Following centrifugation at 20,000 × *g* for 10 min, the supernatant was removed and the precipitates were dissolved in 0.1 M NaOH/1% Triton X-100 overnight at room temperature. The samples were mixed with Ultima Gold scintillation liquid (Perkin Elmer) and myotube protein radioactivity was measured using a Wallac 1409 DSA liquid scintillation counter (Perkin Elmer). Counts were normalized to total genomic DNA, which was extracted using the Allprep DNA/RNA mini kit (Qiagen, Clifton Hill, VIC, Australia) as per the manufacturer’s protocol. DNA was quantified using the NanoDrop 2000 (NanoDrop Products, Wilmington, DE, United States).

### Myotube Diameter

Myotubes were visualized using an Olympus IX70 microscope (Olympus, Mt Waverly, VIC, Australia), and digital images were obtained using a DS-U3 microscope camera and NIS-Elements imaging software (Nikon Instruments Inc., Melville, NY, United States). Approximately 10 myotubes from 10 fields of view were analyzed for myotube diameter from each of the different adenoviral infections.

### RNA Extraction

RNA was extracted from C2C12 myotubes using Tri-Reagent solution (Ambion Inc., Austin, TX, United States) according to manufacturer’s protocol. RNA was extracted from human muscle before and after resistance exercise using the TōTALLY RNA^TM^ Kit (Ambion Inc.). RNA quality and concentrations were determined using the NanoDrop 2000 and Agilent 2100 Bioanalyzer (Agilent Technologies, Palo Alto, CA, United States) for the C2C12 and human muscle samples, respectively. RNA was reverse transcribed to complementary DNA (cDNA) using a high-capacity cDNA reverse transcription kit (Applied Biosystems, Forster City, CA, United States) according to manufacturer’s protocol.

### Semi-Quantitative Real-Time Polymerase Chain Reaction (qRT-PCR)

Individual gene expression analysis was performed by qRT-PCR using a Stratagene Mx3000P QPCR System and the MxPro QPCR software (Stratagene, La Jolla, CA, United States). Cycling conditions for the qPCR consisted of one denaturing cycle at 95°C for 2 min, followed by 40 cycles of denaturing at 95°C for 5 s and annealing at 60°C for 20 s, and elongation at 72°C for 60 s. Each 20 μL reaction contained 0.5 × Power SYBR Green PCR Master Mix (Applied Biosystems), 25 ng of cDNA, and the forward and reverse primers for the gene of interest. Primers were designed using Primer 3^1^ and the sequences crosschecked for gene specificity using a BLAST search^2^. Primers were synthesized by GeneWorks (Adelaide, SA, United States). Primer sequences for the genes amplified in the qRT-PCR are in **Supplementary Table S1**. To compensate for variations in input RNA amounts and efficiency of the reverse transcription, data were normalized to ribosomal protein, 36B4 (also known as RPLPO) mRNA.

### Protein Extraction

Myotubes were lyzed in RIPA buffer (Merck-Millipore, VIC) with 1 μL/mL protease inhibitor cocktail (Sigma-Aldrich) and 10 μL/mL Halt Phosphatase Inhibitor Single-Use Cocktail (Thermo Scientific, Rockford, IL, United States). Protein concentrations were determined using the bicinchoninic acid (BCA) Protein Assay Kit (Thermo Scientific) according to the manufacturer’s protocol, and absorbance was measured at 562 nm on a Synergy 2 Microplate Reader (BioTek, Winooski, VT, United States).

### Western Blotting

Electrophoresis was performed using a 4–12% NuPAGE^®^ Novex Bis-Tris Gel (Invitrogen) in NuPAGE^®^ SDS MOPS Running Buffer (Invitrogen). Protein transfer was performed in a Bjerrum buffer containing 50 mM Tris, 17 mM glycine, and 10% methanol using polyvinylidene difluoride (PVDF) membranes (Merck-Millipore). The membranes were blocked with 5% bovine serum albumin (BSA; Sigma-Aldrich) in PBS for 1 h, and incubated overnight at 4°C with the following primary antibodies diluted in 5% BSA in PBS: PGC-1α (Merck-Millipore); PGC-1β (Novus Biologicals, Littleton, CO, United States); ERRα (Epitomics, Burlingame, CA, United States); glyceraldehyde 3-phosphate dehydrogenase (GAPDH; Sigma-Aldrich); Phospho-Akt (ser473), Akt, Phospho-p70S6k (thr389), p70S6k, Phospho-4E-BP1 (Thr37/46), and 4E-BP1 (Cell Signaling Technology, Danvers, MA, United States). All primary antibodies were diluted 1:1,000, except for GAPDH which was diluted 1:10,000. Following washing, the membranes were incubated for 1 h with either IRDye^®^ 800CW Goat Anti-Rabbit IgG (H + L) (LI-COR Biosciences, Lincoln, NE, United States), or Alexa Fluor^®^ 680 Rabbit Anti-Mouse IgG (H + L) (Invitrogen), diluted 1:5,000 in PBS containing 50% Odyssey Blocking Buffer (LI-COR Biosciences). Proteins were visualized on an Odyssey Infrared Imaging System and densitometry was achieved using Odyssey Application Software 3.0 (LI-COR Biosciences). All blots were normalized to GAPDH protein. Full scans of entire gels are included in **Supplementary Figures S3–S5**.

### Microarray Analysis

The DNA microarray, data transformation, and ANOVA analyses were performed by the Australian Genome Research Facility (AGRF; Parkville, VIC, Australia). The quality and quantity of the RNA extracted from the C2C12 myotubes overexpressed with GFP, PGC-1α, and PGC-1β (a total of six samples for GFP and PGC-1β, and three samples for PGC-1α) were determined with the Agilent Bioanalyzer and RNA 6000 Nano Assay kit (Agilent Technologies). A total of 500 ng of RNA was labeled using the Total Prep RNA amplification kit (Ambion). Labeled cRNA (0.05 μg/μL) was then combined with the GEX-HYB Hybridisation Buffer in a 30 μL final volume and hybridized to the Illumina Mouse-6v2 Expression Beadchip (Illumina Inc., San Diego, CA, United States) overnight at 58°C for 16 h. The chips were washed as outlined in the Illumina manual, coupled with Cy3, and scanned in the Illumina iScan Reader using GenomeStudio software (Illumina Inc.). Raw signal intensity values were subjected to variance stabilization transformation including background correction, log2 transformation, and variance stabilization using the lumiR package of R Bioconductor^3^ (Du et al., 2008; Lin et al., 2008). ANOVA of normalized Illumina probe intensity values was performed using Partek^®^ Genomic SuiteTM software, version 6.6 (Partek Inc., St. Louis, MO, United States). Pairwise comparison of sample groups was performed using ANOVA. Differentially expressed genes were selected by a change in expression level of at least 100% (fold-change > ± 1.5) in compared groups, and a “nominal” ANOVA *p*-value < 0.05.

### Bioinformatics Analysis of Microarray Data

Gene set enrichment analysis (GSEA) was performed using Database for Annotation, Visualization, and Integrated Discovery (DAVID) v6.7^4^ (Huang da et al., 2009a,b) on the differentially expressed gene lists generated from the DNA microarray. Gene Ontology (GO) terms^5^ were identified through DAVID.

### Unbiased, Discriminant Gene Selection

To identify a small subset of genes from the microarray data that could optimally differentiate the response to PGC-1α and PGC-1β overexpression, a combination of diagonal linear discriminant analysis (DLDA) and the signal-to-noise ratio (SNR) statistics (Stegmaier et al., 2004) was used as described previously (Konstantopoulos et al., 2011). A DLDA algorithm generated gene sets that classified arrays into each treatment group. Using a forward step-wise variable selection, sets of genes from the dataset with optimal predictive power were then identified and ranked according to their SNR statistic. To eliminate genes that were co-regulated, Statistical Package for the Social Sciences software (SPSS version 20.0, Fullerton, CA, United States) was used to reduce signature genes to a small subset that were discriminating and displayed divergent expression profiles not highly correlated to each other. The result of these analyses was the identification of a subset of transcripts, similar to a gene expression signature (van de Vijver et al., 2002), which best predict the effect of PGC-1α and PGC-1β overexpression. Seven genes (four upregulated, three downregulated) in this subset that were common to both PGC-1α and PGC-1β regulation were then measured in skeletal muscle samples from individuals following acute resistance exercise via qRT-PCR.

### Resistance Exercise

Eight untrained, but recreationally active, individuals participated in the exercise component of the study. Subject characteristics are presented in **Supplementary Table S2**. Exclusion criteria included resistance training within the past 6 months, medication, or a history of a condition or illness that would present as a health risk during strenuous resistance exercise. All experimental procedures involved in this study were formally approved by the Deakin University Human Research Ethics Committee (EC 18-2004).

### Experimental Design

Each subject in the exercise group completed a familiarization session prior to the experimental protocol. During the session, a 5 RM test was performed for each subject for the leg press, squat (assisted by the Smith machine), and leg extension. 1 RM was then calculated using the Brzycki equation (1 RM = weight lifted (kg)/1.0278 [reps to fatigue × 0.0278]). The familiarization session took place at least 7 days prior to the trial to allow sufficient recovery time before the experimental trial commenced. For the 24 h preceding exercise, and the day of the trial, the subjects consumed a standard diet (20% fat, 14% protein, and 66% carbohydrate) and abstained from alcohol, caffeine, tobacco, and additional exercise. On the morning of the trial, subjects presented to the laboratory in a fasted state. Following 30 min of supine resting, a muscle sample was collected from the vastus lateralis under local anesthesia (xylocaine 1%) by percutaneous needle biopsy technique modified to include suction. Excised muscle tissue from each biopsy was immediately frozen and stored in liquid nitrogen for later analysis. Following 5 min of light cycling for warm-up, subjects completed two sets of 8–12 repetitions of bilateral leg press, squat, and leg extension at 80% 1 RM. This was followed by a third set to voluntary fatigue, also at 80% 1 RM. Subjects had 1 min rest between exercises and 3 min rest between sets. The intensity of this exercise protocol was similar to other studies on resistance exercise (Louis et al., 2007; Buford et al., 2009; Vella et al., 2012). Additional muscle samples were collected at 2, 4, and 24 h following the exercise. Biopsies were collected from separate incisions at least 2 cm distal from previous sites.

### Statistics

Statistical analyses for the cell culture experiments were performed using either a student’s *t*-test, or a two-way ANOVA followed by Tukey’s multiple comparisons test with the adjusted *P*-value set to *P* < 0.05 (GraphPad Software, La Jolla, CA, United States). A one-way repeated measure ANOVA with a Bonferroni adjustment was used for the resistance exercise samples. All data are presented as mean ± standard error of the mean (SEM), unless otherwise stated.

## Results

### PGC-1α and PGC-1β Increase Protein Synthesis and Myotube Diameter in C2C12 Myotubes

**FIGURE 1:**
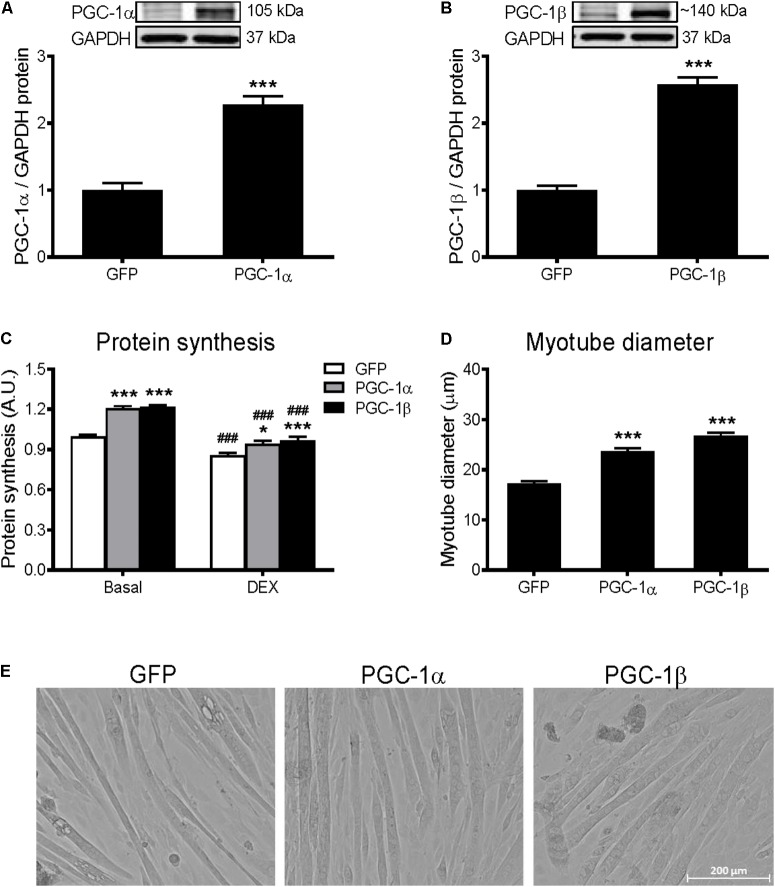
Protein synthesis and myotube diameter in GFP, PGC-1α, and PGC-1β infected C2C12 myotubes. **(A)** PGC-1α and **(B)** PGC-1β protein 72 h after infection with GFP, PGC-1α, and PGC-1β adenoviruses. Bands were normalized to GAPDH protein; *n* = 4 per group. ^∗∗∗^*P* < 0.001 vs. GFP. **(C)** Protein synthesis under basal and dexamethasone (DEX)-treated conditions, when measured for 24 h following 48 h of infection with GFP, PGC-1α, and PGC-1β adenoviruses. *n* = 5–10 per group. ^∗^*P* < 0.05, ^∗∗∗^*P* < 0.001 vs. GFP within the same treatment; ^###^*P* < 0.001 vs. basal within the same condition. **(D)** Average myotube diameter from 10 myotubes per visual field (10 visual fields for each group). ^∗∗∗^*P* < 0.001 vs. GFP. **(E)** Representative images of myotubes infected with GFP, PGC-1α, and PGC-1β adenoviruses for 72 h.

To overexpress PGC-1α and PGC-1β, C2C12 myotubes were infected with adenoviruses expressing GFP (control), PGC-1α, or PGC-1β. PGC-1α and PGC-1β overexpression led to a 2.2-fold and 2.6-fold increase in PGC-1α and PGC-1β protein levels, respectively (**Figures 1A,B**). To investigate the effect of PGC-1α and PGC-1β overexpression on protein synthesis, the incorporation of [^3^H]-tyrosine was measured over 24 h following adenoviral infections. Protein synthesis was measured under basal conditions or with DEX treatment to induce catabolic stress (Long et al., 2001). Overexpression of PGC-1α and PGC-1β increased the basal rate of protein synthesis by ∼20%, respectively, when compared to GFP (**Figure 1C**). DEX treatment decreased basal protein synthesis by ∼15%, while overexpression of PGC-1α or PGC-1β attenuated this effect. Similarly, overexpression of PGC-1α and PGC-1β increased myotube diameter (**Figures 1D,E**).

### PGC-1α and PGC-1β Do Not Increase Protein Synthesis via Activation of Akt/mTOR Signaling

**FIGURE 2:**
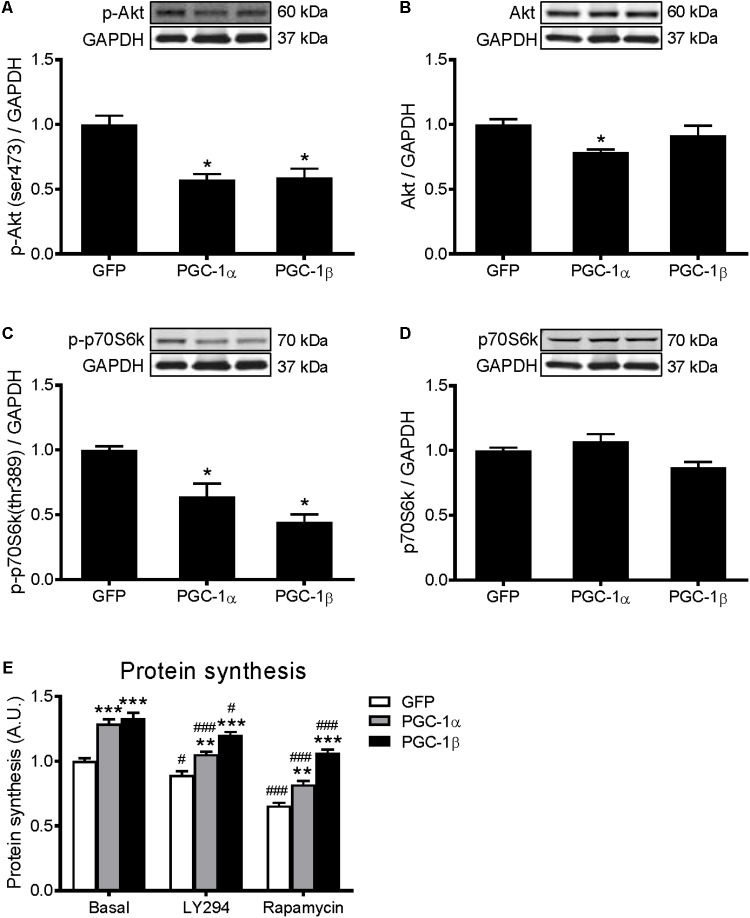
Western blot analysis of Akt and p70S6k proteins in GFP, PGC-1α, and PGC-1β infected C2C12 myotubes. Myotubes were infected with GFP, PGC-1α, or PGC-1β adenoviruses for 48 h, and samples were extracted after 72 h. **(A)** Phospho-Akt (ser473), **(B)** total Akt protein, **(C)** phospho-p70S6k (thr389), and **(D)** total p70S6k protein expression. Samples were harvested after 72 h of infection. Bands were normalized to GAPDH protein. The same control images have been used for **A**,**C**, and **B**,**D**. *n* = 5 per group. ^∗^*P* < 0.01 vs. GFP. **(E)** Protein synthesis in GFP, PGC-1α, and PGC-1β infected C2C12 myotubes, treated with LY294002 (LY294) or Rapamycin and compared to basal conditions. *n* = 6, repeated in three experiments. ^∗∗^*P* < 0.01, ^∗∗∗^*P* < 0.001 vs. GFP within the same treatment; ^#^*P* < 0.05, ^###^*P* < 0.001 vs. control within the same condition.

Activation of Akt/mTOR signaling promotes skeletal muscle protein synthesis via phosphorylation of their downstream substrates, p70S6k and 4E-BP1, enhancers of protein translation initiation and elongation (Bodine et al., 2001; Rommel et al., 2001). To investigate if the Akt/mTOR pathway is regulated by PGC-1α or PGC-1β, protein expression of key proteins in this pathway was measured. Overexpression of PGC-1α and PGC-1β decreased the phosphorylation of Akt (**Figure 2A**) and p70S6k (**Figure 2C**) by 40–50%. There was also a decrease in total Akt protein with PGC-1α overexpression (**Figure 2B**), but no changes in total p70S6k (**Figure 2D**). However, there was no change in the phosphorylated or total levels of 4E-BP1 (**Supplementary Figures S1A,B**). To further investigate whether Akt/mTOR signaling is involved in the PGC-1α- and PGC-1β-induced stimulation of protein synthesis, myotubes were treated with a PI3K inhibitor, LY294002, or an mTOR inhibitor, rapamycin (Rommel et al., 2001; Latres et al., 2005). Compared to the basal group, treatment with LY294002 and rapamycin decreased protein synthesis similarly in the GFP, PGC-1α and PGC-1β-infected myotubes (**Figure 2E**). These results show that both PGC-1α and PGC-1β do not increase protein synthesis via the activation of Akt/mTOR signaling in myotubes.

### ERRα Is Involved in the PGC-1α- and PGC-1β-Induced Increase in Protein Synthesis

**FIGURE 3:**
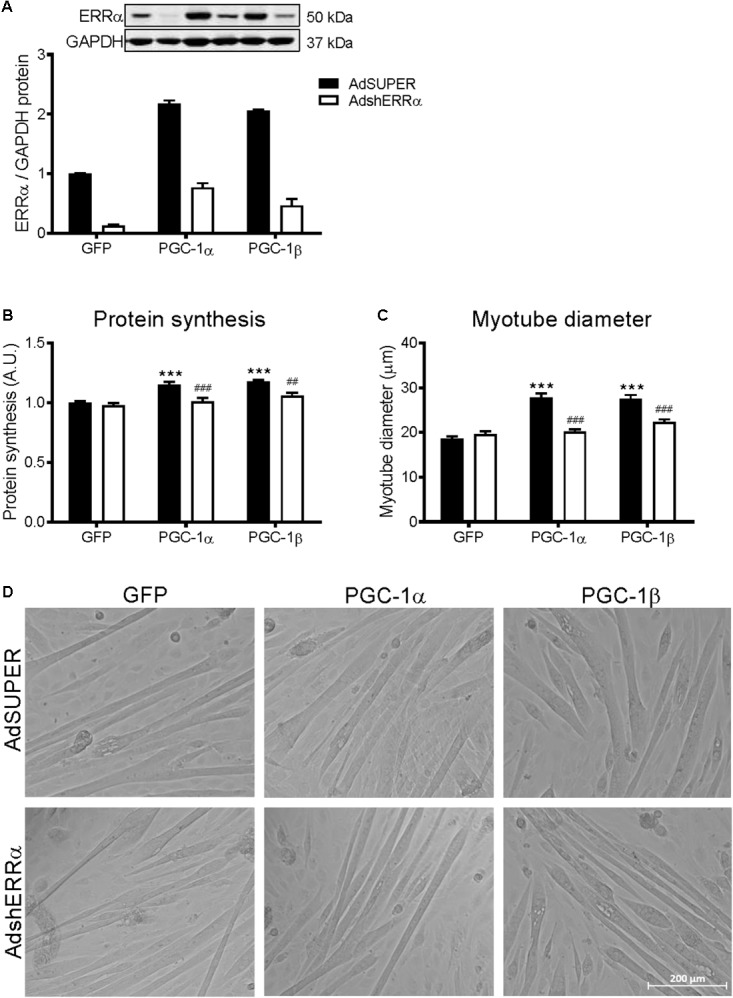
Protein synthesis and myotube diameter in C2C12 myotubes infected with AdshERRα and PGC-1 adenoviruses. Myotubes were infected with either AdSUPER or AdshERRα for 24 h, followed by infection with GFP, PGC-1α, or PGC-1β for a further 48 h. **(A)** ERRα protein, normalized to GAPDH protein. *n* = 3–4 per group. **(B)** Protein synthesis, measured via [^3^H]-tyrosine incorporation for 24 hours after infections. *n* = 6 per group, repeated in three experiments. **(C)** Average myotube diameter from 10 myotubes per visual field (10 visual fields for each group). **(D)** Representative images of GFP, PGC-1α, and PGC-1β infected myotubes, with AdSUPER or AdshERRα. ^∗∗∗^*P* < 0.001 vs. GFP-AdSUPER. ^##^*P* < 0.01, ^###^*P* < 0.001 vs. AdSUPER for each condition. A.U., arbitrary units.

**FIGURE 4:**
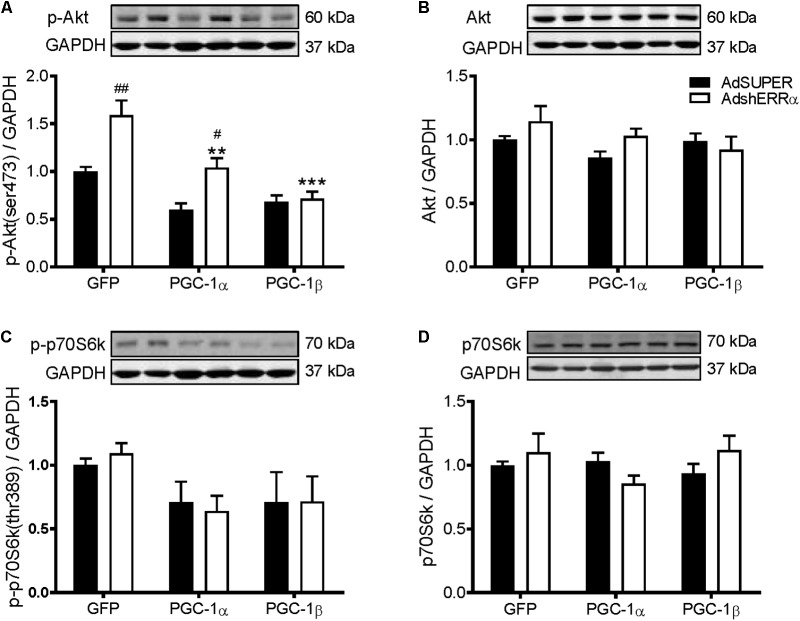
Western blot analysis of Akt and p70S6k proteins in C2C12 myotubes infected with AdshERRα and PGC-1 adenoviruses. Myotubes were infected with either AdSUPER or AdshERRα for 24 h, followed by infection with GFP, PGC-1α, or PGC-1β for a further 48 h. Samples were harvested after 96 h. **(A)** Phospho-Akt (ser473), **(B)** total Akt protein, **(C)** phospho-p70S6k (thr389), and **(D)** total p70S6k protein expression. Bands were normalized to GAPDH protein. The same control images have been used for **A–C**. *n* = 4 per group. ^∗∗^*P* < 0.01, ^∗∗∗^*P* < 0.001 vs. GFP-AdShERRα; ^#^*P* < 0.05, ^##^*P* < 0.01 vs. to AdSUPER for each condition.

Many of the PGC-1α and/or PGC-1β-mediated effects on skeletal muscle also involve their activation of ERRα (Mootha et al., 2004; Schreiber et al., 2004; Shao et al., 2010). To determine if ERRα is involved in the PGC-1α- and PGC-1β-induced increase in protein synthesis, C2C12 myotubes were infected with an adenovirus expressing shRNA for ERRα (AdshERRα) or a control vector (AdSUPER), in combination with GFP, PGC-1α, and PGC-1β adenoviruses. As expected (Kamei et al., 2003; Mootha et al., 2004), PGC-1α and PGC-1β overexpression increased ERRα protein expression (**Figure 3A**). AdshERRα decreased ERRα protein by 87% when compared to AdSUPER and prevented the increase in ERRα protein expression that was seen with PGC-1α and PGC-1β overexpression (**Figure 3A**). AdshERRα had no effect on basal protein synthesis in the GFP-infected myotubes (**Figure 3B**). However, when overexpressed with PGC-1α or PGC-1β, AdshERRα attenuated the PGC-1α- and PGC-1β-induced increase in protein synthesis (**Figure 3B**). Similarly, AdshERRα alone did not affect myotube diameter in the GFP-infected myotubes but ameliorated the PGC-1α- and PGC-1β-induced increase in myotube diameter (**Figures 3C,D**). As it was shown that PGC-1α and PGC-1β reduced the phosphorylation of Akt and p70S6k, their expression was also measured to see if it was affected by knockdown of ERRα. AdshERRα increased the phosphorylation of Akt in the GFP and PGC-1α, but not the PGC-1β infected myotubes when compared to the AdSUPER control (**Figure 4A**). There were no significant changes in total Akt protein, phosphorylated p70S6k, or total p70S6k protein (**Figures 4B–D**), or in phosphorylated or total 4E-BP1 (**Supplementary Figures S1A,B**).

### Constitutively Active ERRα Increases Protein Synthesis and Myotube Diameter

**FIGURE 5:**
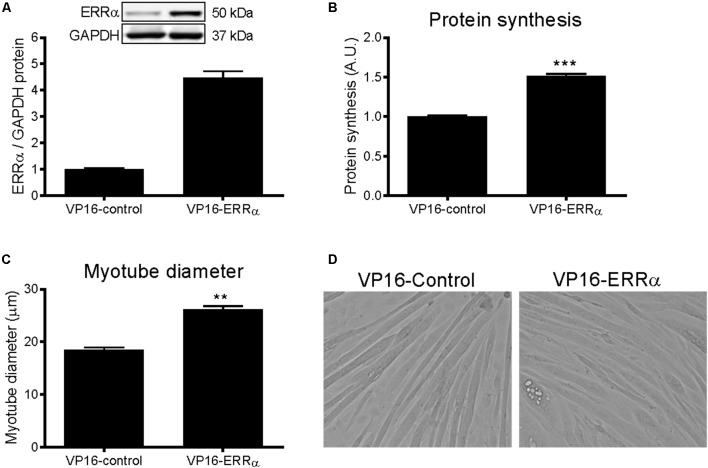
Protein synthesis and myotube diameter in VP16-ERRα infected C2C12 myotubes. **(A)** ERRα protein, normalized to GAPDH protein. *n* = 4 per group. **(B)** Protein synthesis, measured by [^3^H]-tyrosine incorporation for 24 h following 48 h of infection with VP16-control or VP16-ERRα adenoviruses. *n* = 6 per group, repeated in three experiments. **(C)** Average myotube diameter from 10 myotubes per visual field (10 visual fields for each group). **(D)** Representative images of myotubes infected with VP16-control or VP16-ERRα for 72 hours. ^∗∗∗^*P* < 0.001 vs. VP16-control. A.U., arbitrary units.

**FIGURE 6:**
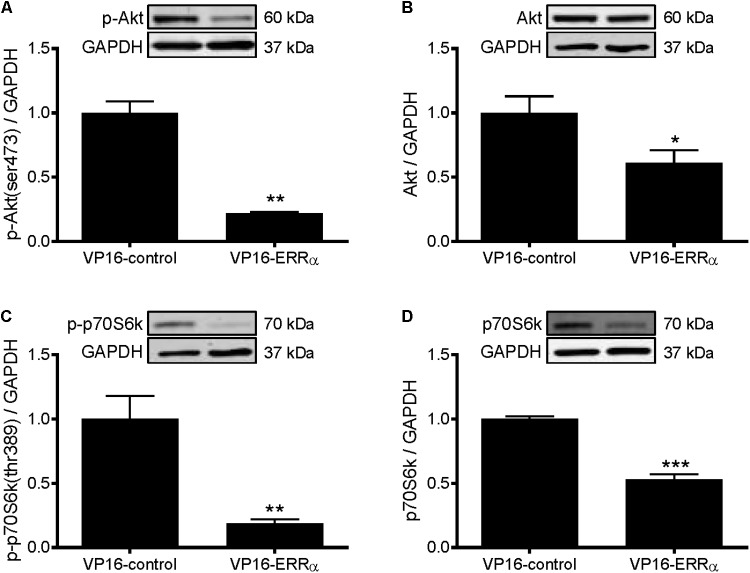
Western blot analysis of Akt and p70S6k proteins in VP16-ERRα infected C2C12 myotubes. Myotubes were infected with VP16-control or VP16-ERRα adenoviruses for 48 h, and samples were extracted after 72 h. **(A)** phospho-Akt (ser473), **(B)** total Akt protein, **(C)** phospho-p70S6k (thr389), and **(D)** total p70S6k protein expression. Bands were normalized to GAPDH protein. The same control images have been used for **A,B**. *n* = 5 per group. ^∗^*P* < 0.05, ^∗∗^*P* < 0.01, ^∗∗∗^*P* < 0.001 vs. VP16-control.

To further support the role of ERRα in positively regulating protein synthesis, C2C12 myotubes were infected with a constitutively active form of ERRα (VP16-ERRα), or its control (VP16-control). VP16-ERRα increased ERRα protein levels by approximately 4.5-fold (**Figure 5A**). VP16-ERRα increased protein synthesis by 52% compared to VP16-control (**Figure 5B**), which resulted in an increase in myotube diameter (**Figures 5C,D**). Lastly, VP16-ERRα decreased Akt and p70S6k phosphorylation (**Figures 6A,C**), and total Akt and p70S6k protein (**Figures 6B,D**) but did not change total or phosphorylated 4E-BP1 (**Supplementary Figure S1B**).

### Microarray and GSEA

**FIGURE 7:**
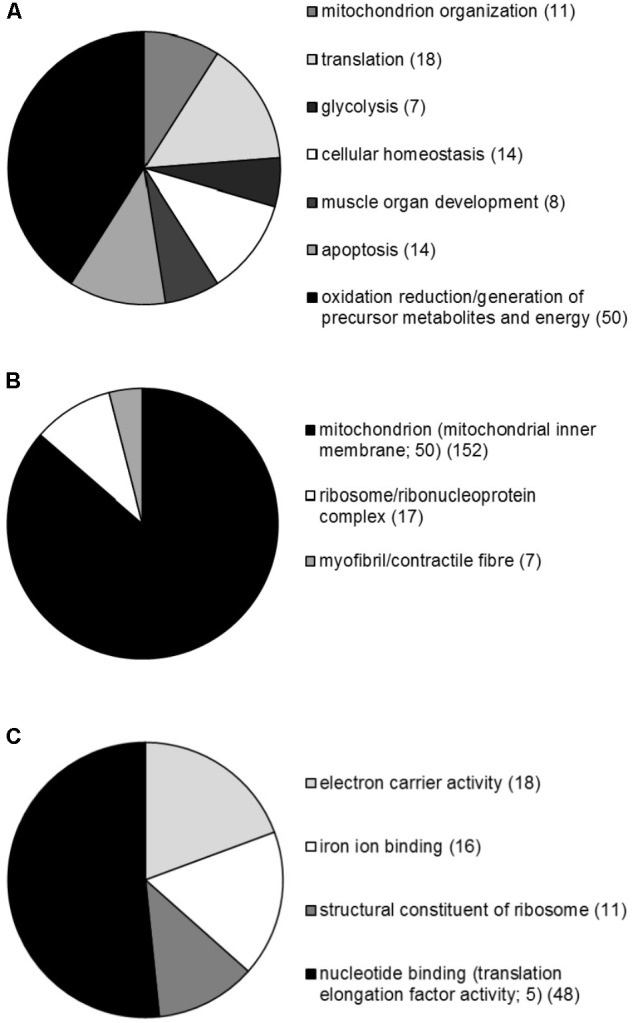
Overview of the GSEA performed on genes commonly regulated by both PGC-1α and PGC-1β in C2C12 myotubes. Proportional representation of gene numbers significantly enriched in GO-related **(A)** biological processes, **(B)** cellular compartment, and **(C)** molecular functions with the number of genes indicated in brackets. The proportion of genes making up the specific sub-groups; CC GO term mitochondrial inner membrane, and MF GO terms translation elongation factor activity are also indicated.

A global overview of genes significantly regulated following PGC-1α and PGC-1β overexpression revealed 1,035 and 3,090 genes, respectively, that displayed at least a 1.5-fold increase or decrease in mRNA levels when compared to the GFP control group (**Supplementary File S1**). As both PGC-1α and PGC-1β enhanced protein synthesis, the genes contributing to protein synthesis activity are likely to be common to both the transcriptional coactivators. Intersect analyses of each gene list (restricted to twofold mRNA change) revealed that 299 genes were commonly regulated by both PGC-1α and PGC-1β (**Supplementary File S2**). These genes were next submitted to GSEA and revealed genes clustered significantly with 94, 37, and 40 biological process, cellular compartment and molecular function GO terms, respectively (**Supplementary File S3**). Predominantly, GO terms relating to mitochondria and metabolism were identified; however, genes were enriched significantly in multiple biological process GO terms including energy metabolism (biosynthetic and catabolic), oxidation–reduction, ion transport and homeostasis, apoptosis, muscle development, and translation. For the cellular compartment, genes associated with GO terms primarily linked with the mitochondrion, in particularly the mitochondrial inner membrane and lumen, in addition to significant associations identified with the ribosome and myofibril/contractile fiber. Furthermore, the molecular function GO terms revealed the majority of gene associations with metabolically related cofactor binding and enzyme activities in addition to translation elongation factor activities and the structural constituent of ribosome. A summary of the biological processes, cellular compartments, and molecular functions linked with GO terms is presented in **Figure 7**. Together, these terms indicate dynamic metabolic, structural, and translational processes occurring in C2C12 myotubes following PGC-1α or PGC-1β overexpression.

### Biased Selection of Genes From the Microarray

**FIGURE 8:**
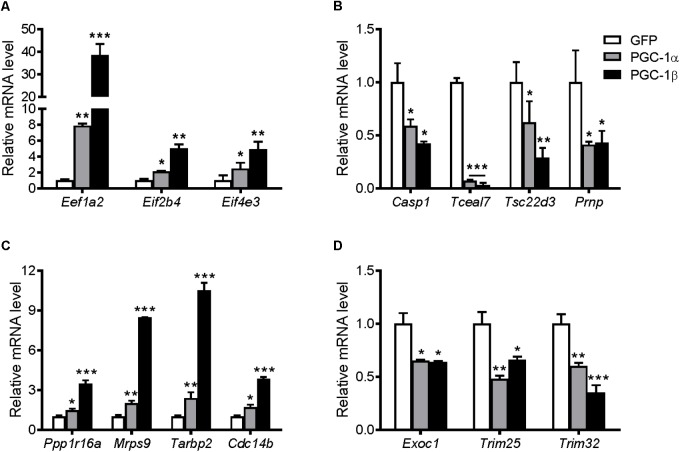
mRNA expression of genes identified from the microarray based on their GO terms involved with protein synthesis, translation, and growth. Myotubes were infected with GFP, PGC-1α, or PGC-1β adenoviruses for 48 h, and samples were extracted after 72 h. mRNA of biasedly selected genes that were **(A)** upregulated and **(B)** downregulated in the microarray. mRNA expression of the genes selected unbiasedly from the microarray that were most significantly **(C)** upregulated and **(D)** downregulated. Values were normalized to 36B4 mRNA expression. *n* = 3, repeated in three experiments. ^∗^*P* < 0.05, ^∗∗^*P* < 0.01, ^∗∗∗^*P* < 0.001 vs. GFP.

To elucidate possible molecular mechanisms by which PGC-1α and PGC-1β regulate protein synthesis, a selection of genes that were upregulated or downregulated by both transcriptional coactivators as revealed by microarray analyses was explored further. The genes and their related GO terms chosen are shown in **Table 1**. Most of the upregulated genes are linked to translation GO terms, while other genes that are linked to apoptosis and cell homeostasis GO terms are primarily downregulated as well as genes associated with protein processing and proteolysis, transcription, or the negative regulation of skeletal muscle tissue development. The expression of these genes was validated via qRT-PCR (**Figure 8**). Overexpression of PGC-1α or PGC-1β led to increased gene expression for eukaryotic translation elongation factor 1 alpha 2 (*Eef1a2*), eukaryotic translation initiation factor 2B, subunit 4 (*Eif2b4*), and eukaryotic translation initiation factor 4E family member 3 (*Eif4e3*) (**Figure 8A**). In contrast, caspase 1 (*casp1*), transcription elongation factor A SII-like 7 (*Tceal7*), TSC22 domain family protein 3 (*Tsc22d3*), and prion protein (*Prnp*) were all decreased in response to PGC-1α and PGC-1β overexpression (**Figure 8B**).

**Table 1:** Genes and their Gene Ontology (GO) terms selected biasedly from the microarray.

	Fold-change to GFP		
Gene	PGC-1α	PGC-1β	GO terms
Eef1a2	10.9	19.6	GO:0006412: translation GO:0043067: regulation of programmed cell death
Eif2b4	1.6	2.6	GO:0006412: translation GO:0042592: homeostatic process
Eif4e3	1.9	1.9	GO:0006412: translation GO:0042592: homeostatic process
Casp1	-1.8	-2.2	GO:0043065: positive regulation of apoptotic process;
Tsc22d3	-1.5	-2.5	GO:0048642: negative regulation of skeletal muscle tissue development
Prnp	-1.6	-2.6	GO:0042592: homeostatic process GO:0043067: regulation of programmed cell death
Tceal7	-4.6	-8.4	GO:0006351: transcription, DNA-dependent

### Unbiased Selection of Genes From Microarray Data

Statistical analyses of the entire microarray data set revealed seven discriminant genes significantly regulated (*P* < 0.05) by PGC-1α and/or PGC-1β (**Table 2**). The expression of these targets was validated via qRT-PCR (**Figure 8**). Overexpression of PGC-1α or PGC-1β increased the mRNA of protein phosphatase 1, regulatory subunit 16A (*Ppp1r16a*), mitochondrial ribosomal protein sub-unit 9 (*Mrps9*), TAR HIV-1 RNA binding protein 2 (*Tarbp2*), and cell division cycle 14 homolog B (*Cdc14b*) (**Figure 8C**). In contrast, exocyst complex component 1 (*Exoc1*), tripartite motif-containing protein 25 (*Trim25*), and tripartite motif-containing protein 32 (*Trim32*) were downregulated (**Figure 8D**). Although microarray analyses revealed *Trim25* and *Trim32* as regulated separately by PGC-1β and PGC-1α, respectively, the expression of these genes was reduced in response to both PGC-1α and PGC-1β overexpression when measured via qRT-PCR.

**Table 2:** Microarray profile of the most significantly regulated genes by both PGC-1α and PGC-1β overexpression.

Gene	GenBank accession no.	Direction vs. GFP	PGC-1α *P*-value	PGC-1β *P*-value
Ppp1r16a	NM_033371.2	Up	0.001	5.77E-11
Mrps9	NM_023514.3	Up	6.61E-06	4.35E-12
Tarbp2	NM_009319.1	Up	9.03E-04	3.30E-11
Cdc14b	NM_172587.2	Up	0.018	2.76E-10
Exoc1	NM_027270.1	Down	8.04E-05	6.35E-12
Trim25^∗^	NM_009546.2	Down		5.15E-09
Trim32^∗∗^	NM_053084.1	Down	0.001	

^∗^*PGC-1β only; ^∗∗^PGC-1α only*.

### Regulation of Genes in a Model of Muscle Hypertrophy

**FIGURE 9:**
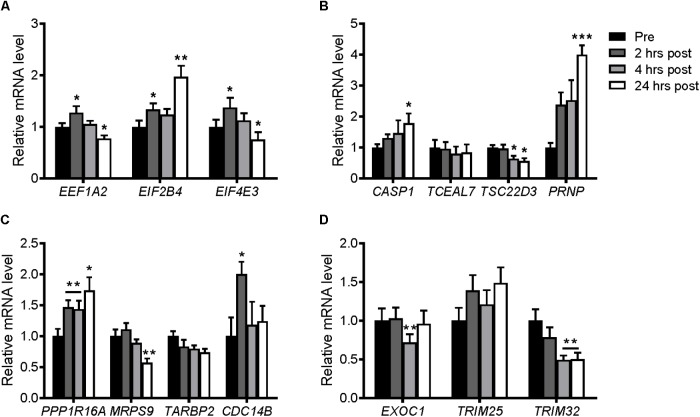
mRNA expression of genes selected from the microarray in skeletal muscle after an acute bout of resistance exercise in humans. Muscle samples were taken before and 2, 4, and 24 h after resistance exercise. **(A)** Upregulated genes and **(B)** downregulated genes selected biasedly; and **(C)** upregulated genes and **(D)** downregulated genes selected unbiasedly from the microarray. Values were normalized to 36B4 mRNA expression. *n* = 8. ^∗^*P* < 0.05, ^∗∗^*P* < 0.01, ^∗∗∗^*P* < 0.001 vs. Pre-exercise (Pre).

Resistance exercise in the young and elderly increase protein synthesis rates (Yarasheski et al., 1993). Similar to previous studies, PPARGC1A mRNA was increased transiently at 2 and 4 h following resistance exercise (Deldicque et al., 2008), and then reduced after 24 h (**Supplementary Figure S2A**). In contrast, PPARGC1B mRNA was decreased at 4 and 24 h post-exercise (**Supplementary Figure S2B**). This was associated with significant increases in EEF1A2, EIF2B4, and EIF4E3 genes at 2 h post-exercise (**Figure 9A**). After 24 h, the expression of EIF2B4 increased further, while EEF1A2 and EIF4E3 mRNA expression returned to, or was slightly below, pre-exercise levels. Of the selected genes that were downregulated in the microarray, only TSC22D3 was decreased following resistance exercise, while CASP1 and PRNP were increased 24 h post-exercise (**Figure 9B**). Of the genes selected unbiasedly from the microarray, PPP1R16A and CDC14B were also increased following resistance exercise (**Figure 9C**), while EXOC1 and TRIM32 were decreased (**Figure 9D**).

## Discussion

Understanding the molecular mechanisms that regulate protein synthesis in skeletal muscle is essential for developing therapeutic interventions to combat muscle atrophy. PGC-1α and PGC-1β are downregulated in many models of muscle atrophy characterized by protein degradation processes greater that protein synthesis, and potentially by mitochondrial dysfunction. Here, we demonstrate that PGC-1α or PGC-1β overexpression increases protein synthesis and myotube diameter, independent of Akt and mTOR signaling, but requiring ERRα. Interestingly, PGC-1α or PGC-1β overexpression was able to restore protein synthesis to control levels following treatment with DEX, a catabolic agent known to reduce protein synthesis and myotube diameter. Whether PGC-1α or PGC-1β overexpression can restore myotube diameter following DEX treatment remains to be determined but warrants future investigation to further understand their role in regulating myotube hypertrophy. A similar cell culture model to the current study previously observed that PGC-1α and PGC-1β attenuated protein degradation, but with no effect on protein synthesis (Brault et al., 2010), although protein turnover was measured for only 2 h following 24 or 48 h of adenoviral infection. The results from the present study, however, show that overexpression of PGC-1α or PGC-1β increased protein synthesis throughout the 24 h following a 48 h adenoviral infection; an effect observed under both basal and DEX-treated conditions. This finding suggests that protein synthesis may need to be measured over a longer period of time in order to detect effects by PGC-1α or PGC-1β overexpression. Alternatively, variable levels of PGC1 protein overexpression between the studies may account for the differences. Brault and others also normalized their protein synthesis values against total protein content, which may off-set any increase in protein synthesis from PGC1 overexpression. Interestingly, here and in other studies (Mortensen et al., 2007; Mathai et al., 2008), human PGC-1β levels in skeletal muscle were decreased following resistance training. However, transgenic mouse studies have reported PGC1β overexpression to replicate an exercise training response in sedentary mice (Lee et al., 2017). Currently, it is unclear of the impact of reduced PGC-1β levels in exercised human muscle.

The Akt and mTOR signaling pathways are well known regulators of protein synthesis in skeletal muscle (Bodine et al., 2001; Rommel et al., 2001). In this study, however, PGC-1α and PGC-1β decreased Akt and p70S6k phosphorylation; the latter activated by mTOR. Furthermore, Akt or mTOR activity inhibited by LY294002 and rapamycin, respectively, was not required for PGC-1α or PGC-1β to increase protein synthesis, nor was increased phosphorylation of 4E-BP1, suggesting the activation of alternate protein translation pathways. Many of the PGC-1α- and PGC-1β-mediated effects in skeletal muscle require ERRα transcriptional activity (Mootha et al., 2004; Schreiber et al., 2004; Shao et al., 2010), and as shown here, knockdown of ERRα prevented the PGC-1α- and PGC-1β-induced increase in protein synthesis and myotube diameter and with no effect during basal conditions suggesting that ERRα may only be involved in the regulation of protein synthesis when stimulated by PGC-1α or PGC-1β. These observations support the role for ERRα as a latent transcription factor, which is only active when partnered with coactivators such as PGC-1α and PGC-1β (Schreiber et al., 2003). The effects of ERRα on protein synthesis were further supported by expression of a constitutively active ERRα (VP16-ERRα) resulting in a large increase in protein synthesis. Other than the roles ERRα plays in muscle repair and regeneration (LaBarge et al., 2014), our finding of ERRα’s promotion of protein synthesis in skeletal muscle cells is novel and the mechanism of how it occurs requires further study. Insight into this mechanism is taken potentially from studies which have already observed a molecular cross-talk between mTOR and ERRα in other cell types and conditions. These include synergism observed between rapamycin and inhibition of ERRα during lipid metabolic activities in fatty liver (Chaveroux et al., 2013), T cell activation (Michalek et al., 2011), and following induction of tumorigenesis in oral squamous cell carcinoma cells (Gan et al., 2017). Similar to PGC-1α and PGC-1β overexpression, the phosphorylation and expression of Akt and p70S6k were inhibited by VP16-ERRα providing additional evidence that PGC-1α and PGC-1β do not increase protein synthesis via activation of Akt/mTOR signaling. The lack of Akt/mTOR activation measured here may reflect the longer-term impact of PGC-1α, PGC-1β, and VP16-ERRα overexpression as endpoint measurements were made 72 h post-viral infection. This time-frame may allow for alternate molecular adaptations to promote protein synthesis including mitochondrial and ribosomal biogenesis as indicated in the GSEA findings.

To highlight novel PGC-1α- and PGC-1β-regulated targets that may influence muscle protein synthesis and to extend our knowledge of the molecular adaptations occurring with PGC-1α or PGC-1β overexpression, large-scale gene expression profiling using microarray analyses was performed. The GSEA revealed GO terms associated with biosynthetic and catabolic processes, mitochondria, muscle tissue development, ribosomal and translational activities, supporting the concept of active protein synthesis and metabolic adaptations occurring in the C2C12 myotubes. Enhanced protein synthesis in response to PGC-1α or PGC-1β overexpression may be in part due to the generation of mitochondrial- and metabolic-related proteins, which support known roles for PGC-1α and PGC-1β in mitochondrial biogenesis and function (Wu et al., 1999; St-Pierre et al., 2003; Schreiber et al., 2004; Soriano et al., 2006; Benton et al., 2008; Liesa et al., 2008; Espinoza et al., 2010; Shao et al., 2010). Interestingly, other genes identified in our microarray screen may also be linked to protein synthesis, but independent of mitochondrial biogenesis (**Supplementary File S2**). One such gene is the N-myc downstream-regulated gene 2 (*Ndrg2*), which we previously identified as a target of PGC-1a/ERRa transcriptional regulation (Foletta et al., 2013). The suppression or overexpression of *Ndrg2* increased and decreased protein synthesis, respectively, without altering the expression of gene markers of mitochondrial biogenesis (Foletta et al., 2013). Therefore, these GO terms further indicate roles for PGC-1α and PGC-1β in protein synthesis related to both mitochondrial and non-mitochondrial processes in C2C12 myotubes.

Supporting their potential role in protein synthesis, PGC-1α and PGC-1β overexpression increased the mRNA of elongation and translation initiation factor genes including *Eef1a2*, *Eif2b4*, and *Eif4e3*. Interestingly, these were genes also increased in skeletal muscle following resistance exercise. Deletion of *Eef1a2* due to a spontaneous mutation has been shown previously to result in severe muscle wasting and weakness in mice (Chambers et al., 1998; Newbery et al., 2005), and is a key factor in muscle protein translation following denervation (Doig et al., 2013) further suggesting a role for *Eef1a2* in muscle mass control. A number of the discriminant genes unbiasedly selected also shows links to the regulation of protein synthesis and atrophy. For example, *Trim32* encodes an E3 ubiquitin ligase that degrades thin myofibrillar contractile proteins during muscle atrophy (Cohen et al., 2012), and in humans, is upregulated in muscle of patients with DMD (Assereto et al., 2016). PGC-1α and PGC-1β are known to attenuate skeletal muscle protein degradation by downregulating other ubiquitin ligases, Atrogin-1 and Muscle Ring Finger-1 (Brault et al., 2010) and, therefore, may also target *Trim32* is this process. Furthermore, TARBP2 contributes the control of protein synthesis during spermatogenesis (Braun, 2000), but whether it does in skeletal muscle processes remains to be investigated. Additional studies are required to determine the interaction between PGC-1 proteins and these genes, and their effect on protein synthesis in skeletal muscle.

## Conclusion

This study has shown for the first time that the transcriptional coactivators, PGC-1α and PGC-1β, can increase protein synthesis and myotube hypertrophy in C2C12 myotubes; a response independent from the Akt and mTOR signaling pathways, but dependent on ERRα. While the large-scale identification of novel downstream targets regulated by PGC-1α and PGC-1β provides insight into how these coactivators regulate skeletal muscle growth and metabolic adaptations, the challenge is now to dissect the interplay of these targets to help promote the anabolic effects of PGC-1α and PGC-1β.

## Ethics Statement

All subjects gave written informed consent in accordance with the Declaration of Helsinki.

## Author Contributions

EB, PS, NK, DC-S, AK, and AR designed the study. EB, CW, PDG, and PS performed the experiments. VF and AS the performed bioinformatic and statistical analyses. EB and AR analyzed and interpreted the data. EB, VF, and AR wrote the manuscript.

## Conflict of Interest Statement

The authors declare that the research was conducted in the absence of any commercial or financial relationships that could be construed as a potential conflict of interest.

## Footnotes

1 www.primer3.sourceforge.net

2 https://blast.ncbi.nlm.nih.gov/Blast.cgi

3 www.Bioconductor.org

4 http://david.abcc.ncifcrf.gov

5 http://www.geneontology.org
