# Supplementary material for: PGC-1α and PGC-1β Increase Protein Synthesis via ERRα in C2C12 Myotubes
Source: Front Physiol. 2018 Sep 25;9:1336. doi: 10.3389/fphys.2018.01336 (PMC6190860; doi:10.3389/fphys.2018.01336)
Supplement: Supplementary file 4 [file Data_Sheet_1.pdf]

PGC-1α and PGC-1β increase protein synthesis via ERRα in C2C12 myotubes

Erin L. Brown[1§*], Victoria C. Foletta[1], Craig R. Wright[1], Patricio V. Sepulveda[1], Nicky Konstantopoulos[2], Andrew Sanigorski[2], Paul Della Gatta[1], David Cameron-Smith[3], Anastasia Kralli[4], Aaron P. Russell[1]

***Correspondence:** Dr. Erin Brown**:** e-mail: erin.brown@sund.ku.dk

Supplementary Data

The datasets for this study can be found in Supplementary Files 1, 2 and 3.

Supplementary Figures and Tables

Supplementary Figures

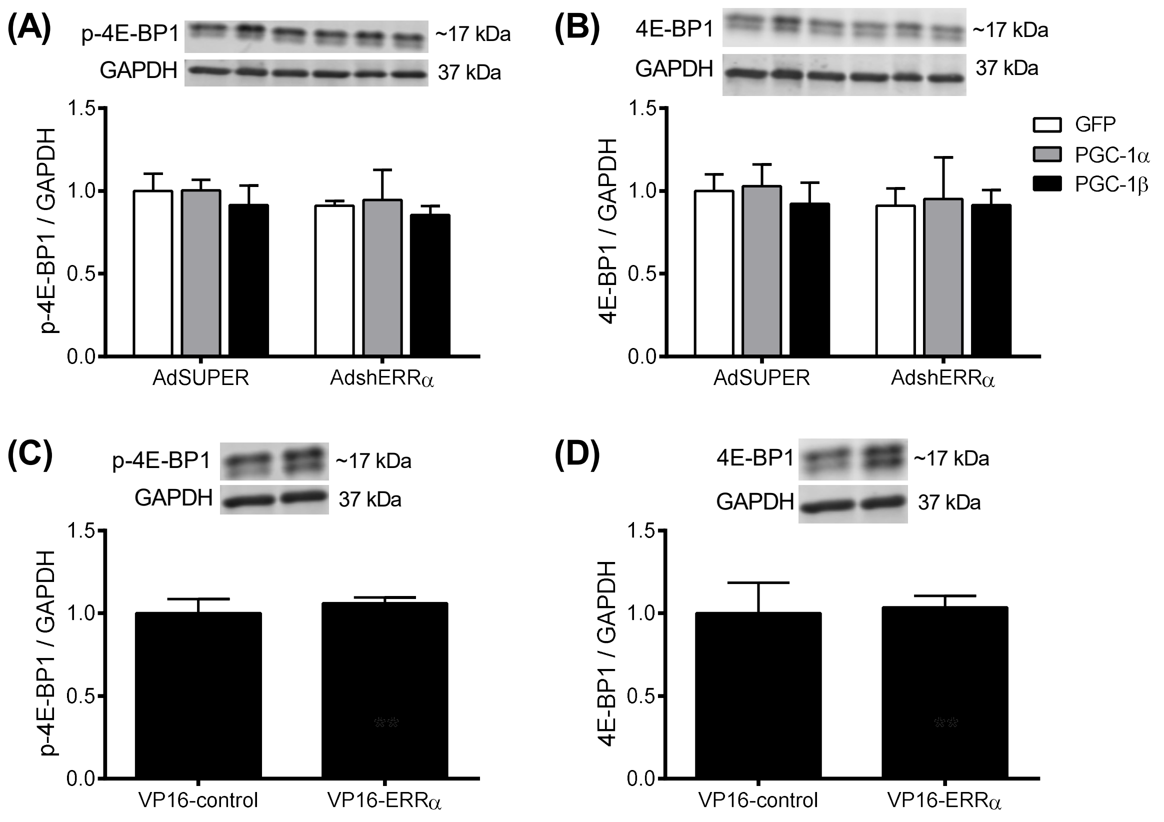

Supplementary Figure 1. Phospho and total 4E-BP1 protein expression in C2C12 myotubes infected with shERRα and PGC-1 or VP16-ERRα adenoviruses. (A) p-4E-BP1, and (B) 4E-BP1, in myotubes infected with either AdSUPER or AdshERRα for 24 hours, followed by infection with GFP, PGC-1α or PGC-1β for a further 48 hours. Samples were harvested after 96 hours. (C) p-4E-BP1, and (D) 4E-BP1, in myotubes infected with VP16-control or VP16-ERRα adenoviruses for 48 hours. Samples were harvested after 72 hours. Bands were normalized to GAPDH protein; n = 3 per group.

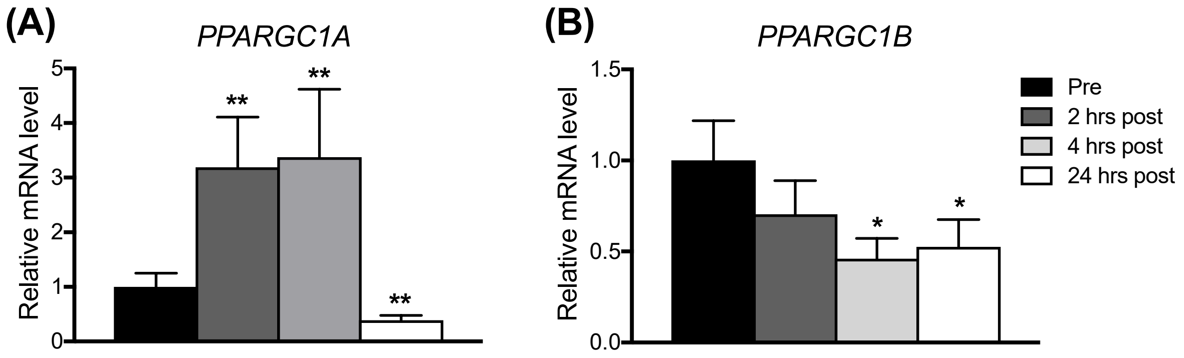

Supplementary Figure 2. PPARGC1A and PPARGC1B mRNA expression in skeletal muscle after an acute bout of resistance exercise in humans. (A) PPARGC1A mRNA, and (B) PPARGC1B mRNA when measured prior to and 2, 4 and 24 hours after resistance exercise. Values were normalized to 36B4 mRNA expression. n = 8. *P < 0.05, **P < 0.01 vs. Pre-exercise.

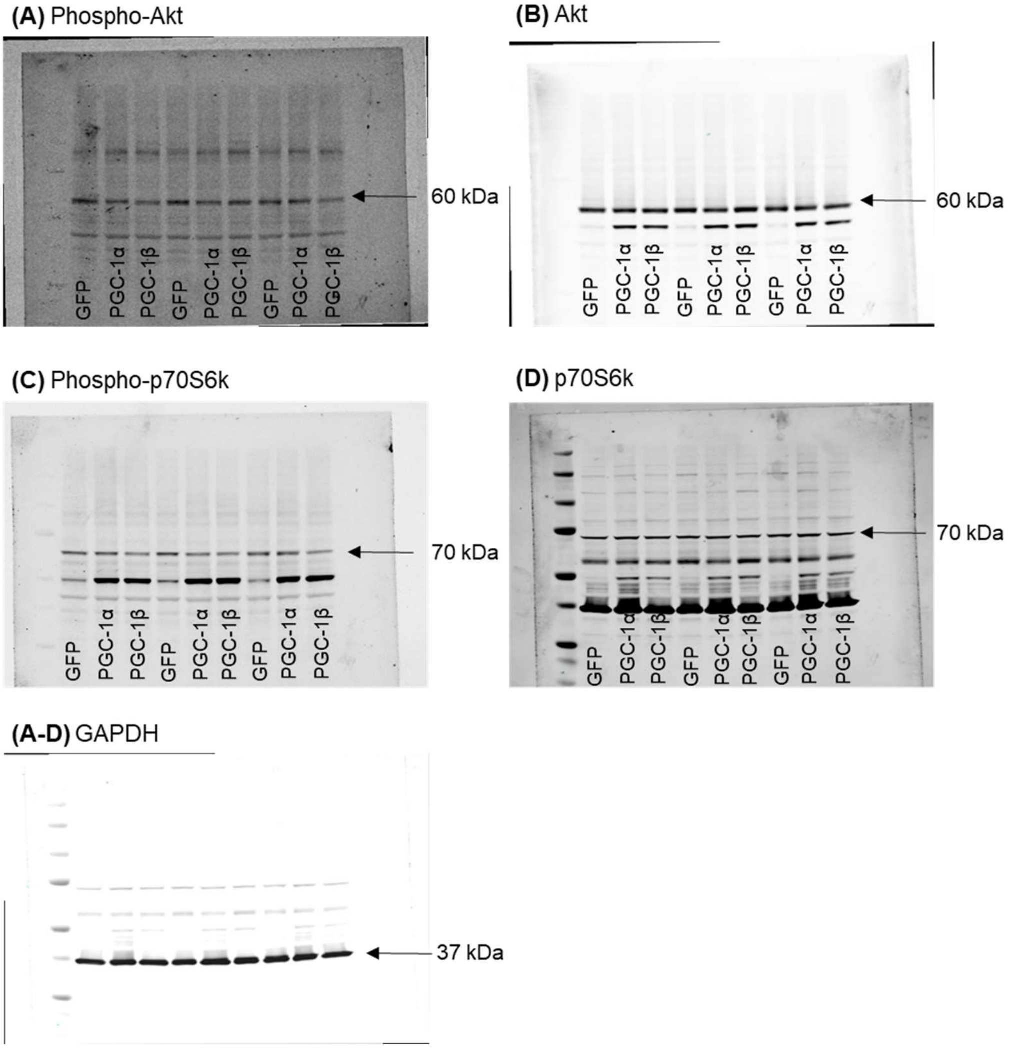

Supplementary Figure 3. Full scans of western blots displayed in Figure 2**.**

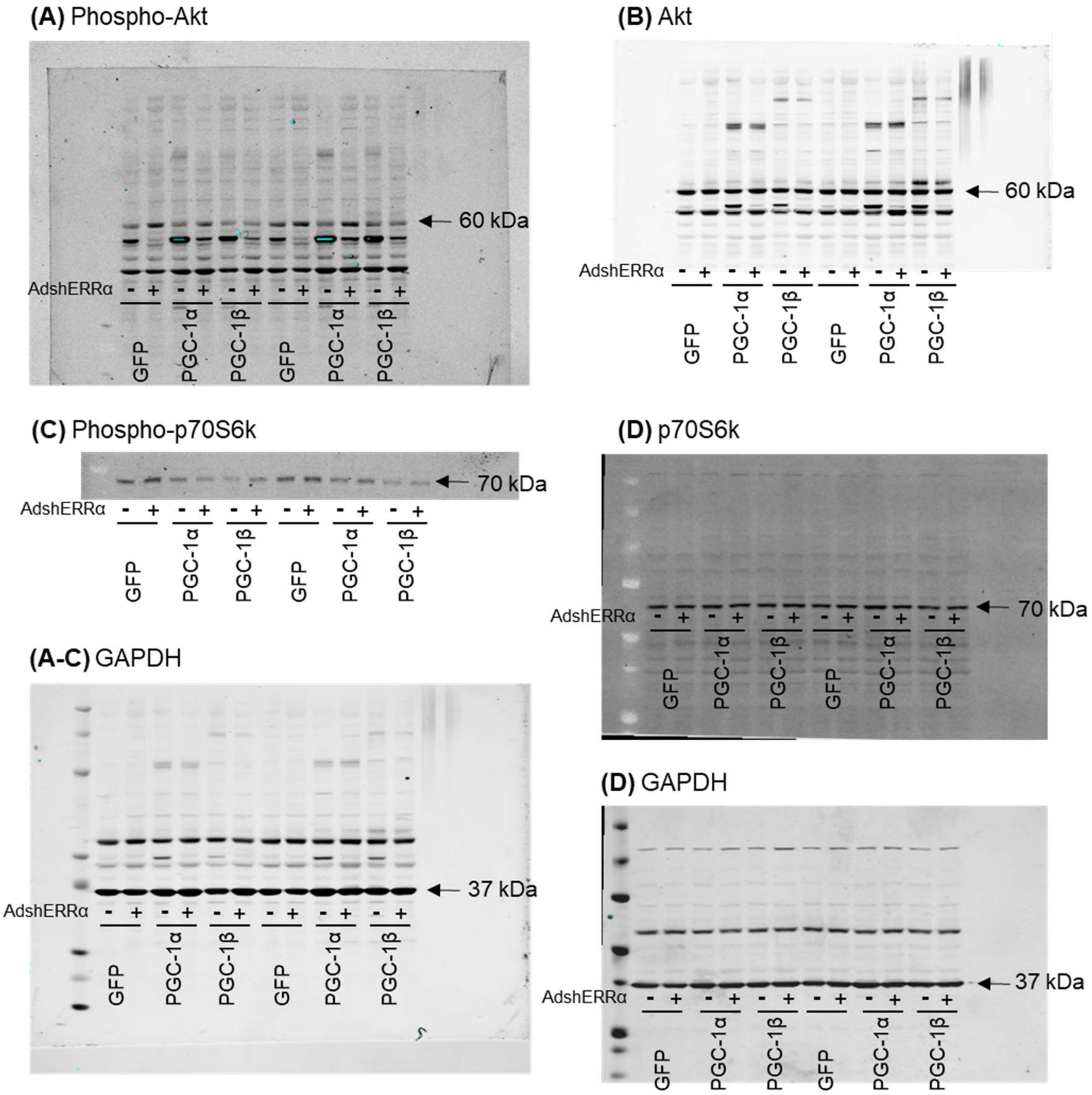

Supplementary Figure 4. Full scans of western blots displayed in Figure 4**.**

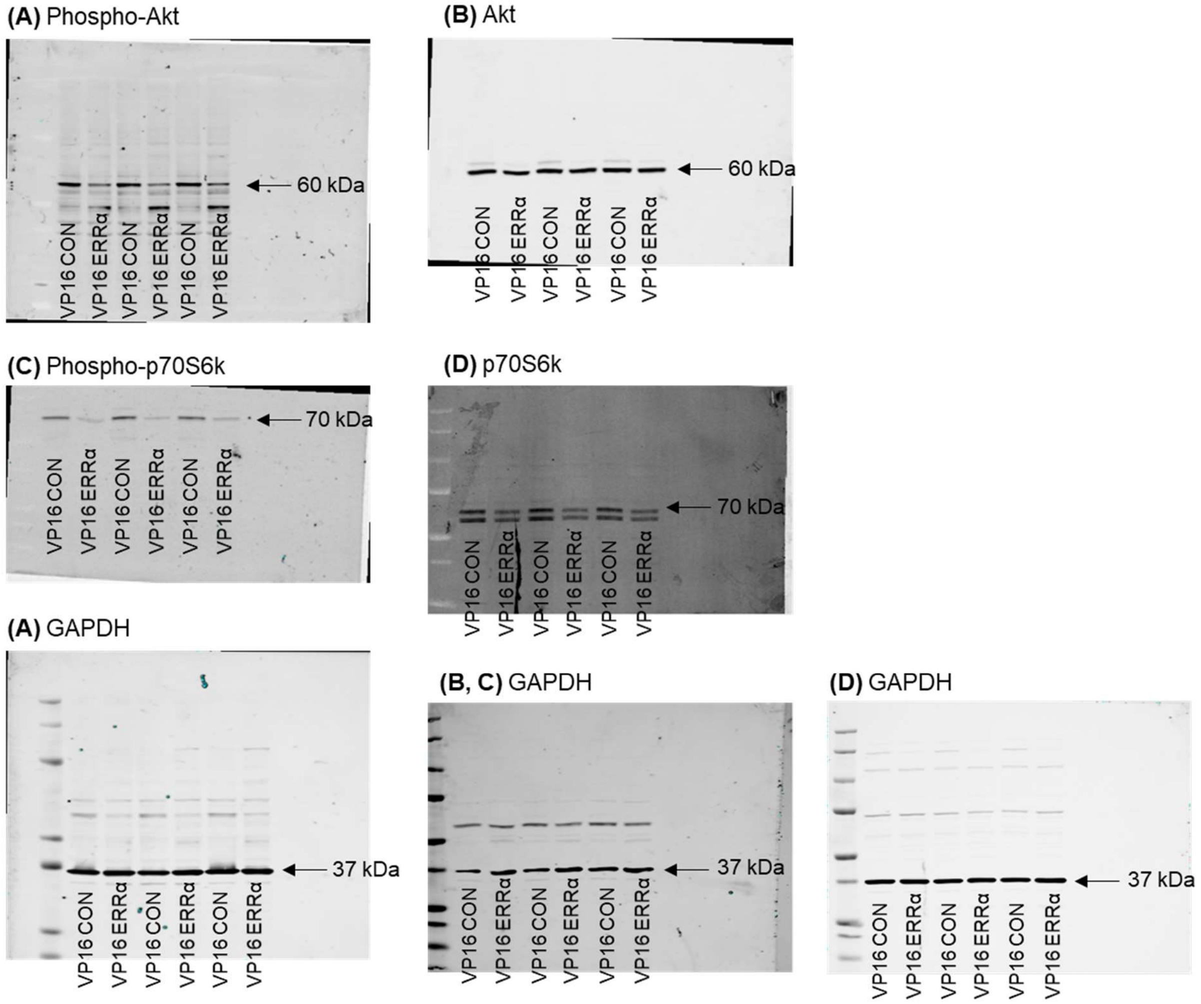

Supplementary Figure 5. Full scans of western blots displayed in Figure 6**.**

Supplementary Tables

Supplementary Table 1. Mouse and Human Primers used in the RT-PCR

Primer	GenBank accession no.	Forward Primer (5'-3')	Reverse Primer (5'-3')
h-Ppargc1a	NM_013261.3	TCAGTCCTCACTGGTGGACA	TGCTTCGTCGTCAAAAACAG
h-Ppargc1b	NM_133263.3	CTGCTGGCCCAGATACACTGA	ATCCATGGCTTCATACTTGCT
m-Ppp1r16a	NM_033371.2	GCTGCACATCGCCGCTGCTA	GGCCCGCAGGAGTGCATCTT
m-Mrps9	NM_023514.3	CACGGCACAGGCGGTCGTTT	CAAGCGTATGGCGCCTGCCT
m-Tarbp2	NM_009319.1	CAGCTGCACTGGTCAGGGCC	GTGCAGCCTGGCCCACGATT
m- Cdc14b	NM_172587.2	GCGTGAAGAAGAGCCGCAGC	ACCATGGCCAGATTGAGTGGTCC
m-Exoc1	NM_027270.1	CACCACGTGTGGACCGCACA	TCGCCACGGCGTTCAGCATT
m-Trim25	NM_009546.2	CCCGAGCATGGCGAGTGCAT	GCTGGTCTCTGCTGCGTCGA
m-Trim32	NM_053084.1	GTGGACTCGCGTCGGAGCTG	GGGCAGCGGACGCCATTGAT
m-Eef1a2	NM_007906.2	CAGTTCACCTCTCAGGTTATCATCCT	GCCGTGTGACAGTCGATGAC
m-Eif2b4	NM_001127356.1	CTTCTCCCACCTGCCTCAGTA	CAGAGGATGGGATGCTCATGTA
m-Eif4e3	NM_025829.4	ACCATTGGAGAGCAGTTCACAGA	TCACGCTGACTCCGATGATC
m-Casp1	NM_009807.2	AAAAGCCCAGAAGTTATGGAAAGA	GACGTGTACGAGTGGTTGTATTCAT
m-Tceal7	NM_001127169.1	AACCCTGCGGAGCACTTG	TGAAGCAACCTCTGTCTGAAATTG
m-Tsc22d3	NM_001077364.1	TTCTCCATCTCCTTCTTTTCTTCTCT	CTCCGGAGGCACTGTTATCC
m-Prnp	NM_011170.2	CACCGAGACCGATGTGAAGA	GGTACTGGGTGACGCACATCT
m-36B4	NM_007475.5	TTGTGGGAGCAGACAATGTG	AGTCCTCCTTGGTGAACACG
h-Ppp1r16a	NM_032902.5	ACGCCCCTTGATGTGTGCGG	GAGGGCGTCGTGCTTGTGCT
h-Mrps9	NM_182640.2	GCCCTGTGTGTCCTACGGCG	CAGAGGCCTTGCTTCCGGGC
h-Tarbp2	NM_134323.1	TCAGCAGTCTGAGTGCAACC	GTGCCACTCCCAATCTCAAT
h-Cdc14b	NM_003671.3	TCGACCTCGCCGGGTGTGAA	ACACGTCGTCCTGGGGGTCC
h-Exoc1	NM_018261.3	AGGCCTGCACCAATGCTGCT	CTGGCCAGTCTCCGGGCAAA
h-Trim25	NM_005082.4	CGACCTGGAGGCCACCCTGA	CCGCACATCCTGCTGCCTGT
h-Trim32	NM_012210.3	GGAGGCCACAGCGTCTGCTG	GGCAACCACTTCCTCCGGGC
h-Eef1a2	NM_001958.3	CGTGGGCGTGAACAAAATG	TCGTAGCGCTTCTCGCTGTA
h-Eif2b4	NM_172195.3	CCGGTGTATTGCCCTGCTT	CATTAGGCGGTGTTGTGTAATCC
h-Eif4e3	NM_001134651.1	GCAGCAGATGATGAAGTAATAGGAGTT	AGTGCCCATCTGTTCTGTAGGGA
h-Casp1	NM_001223.4	CTGCTCTTCCACACCAGATAATGT	TCCAATAAAAACAGAGCCCATTG
h-Tceal7	NM_152278.3	CGCCCGTATGGAGAATTTGA	GAAGCAGCCTCTGTCTAAAATTCC
h-Tsc22d3	NM_198057.2	TGGCCATAGACAACAAGATCGA	TCACAGCATACATCAGATGATTCTTC
h-Prnp	NM_000311.3	ATGGAGCGCGTGGTTGA	GGCCTGAGATTCCCTCTCGTA
h-36B4	NM_001002	GTGATGTGCAGCTGATCAAGACT	GATGACCAGCCCAAAGGAGA

m = mouse, h = human.

Supplementary Table 2. Characteristics of subjects in the resistance exercise study.

**Age (years)**	22.0 ± 05
**Height (m)**	1.79 ± 0.05
**Body mass (kg)**	83.3 ± 19.1
**BMI ($kg.m^{-2}$)**	24.1 ± 0.1
